# Transmission Electron Microscopy (TEM) Observations of Female Oocytes From *Nilaparvata lugens* (Hemiptera: Delphacidae): Antibiotic Jinggangmycin (JGM)-Induced Stimulation of Reproduction and Associated Changes in Hormone Levels

**DOI:** 10.1093/jee/tow085

**Published:** 2016-05-31

**Authors:** Bing Xu, Lin-Lin You, You Wu, Jun Ding, Lin-Quan Ge, Jin-Cai Wu

**Affiliations:** School of Plant Protection, Yangzhou University, Yangzhou 220059, P.R. China (184389952@qq.com; 1532032890@qq.com; 245841916@qq.com; 2312172062@qq.com; lqge1027@163.com; jincaiwu1952@sina.com)

**Keywords:** jinggangmycin, *Nilaparvata lugens*, reproduction, oocyte, hormone level

## Abstract

Previous studies have demonstrated that the agricultural antibiotic jinggangmycin (JGM) stimulates reproduction in the brown planthopper *Nilaparvata lugens* Stål and that the stimulation of brown planthopper reproduction induced by JGM is regulated by the fatty acid synthase (*FAS*) and acetyl-CoA carboxylase (*ACC*) genes. However, a key issue in the stimulation of reproduction induced by pesticides involves the growth and development of oocytes. Therefore, the present study investigated oocyte changes via transmission electron microscopy (TEM) and changes in hormone levels (juvenile hormones (JH) and 20-hydroxyecdysone (20 E)) in JGM-treated females. TEM observations showed that the size of the lipid droplets in the oocytes of JGM-treated females, compared with those in the oocytes of the control females, significantly reduced by 32.6 and 29.8% at 1 and 2 d after emergence (1 and 2 DAE), respectively. In addition, the JH levels of JGM-treated females at 1 and 2 DAE were increased by 49.7 and 45.7%, respectively, whereas 20 E levels decreased by 36.0 and 30.0%, respectively. We conclude that JGM treatments lead to substantial changes in lipid metabolism, which are directly and indirectly related to stimulation of reproduction of brown planthopper together with our previous findings.

The brown planthopper, *Nilaparvata lugens* Stål, is not only the most serious pest in Asia but also a classic example of a pest exhibiting pesticide-induced resurgence. Potential causes of resurgence occurrence mainly involve in the mortality toward beneficial organisms and stimulation of reproduction of brown planthopper caused by pesticides. Intensive studies have been conducted on the pesticide-induced stimulation of reproduction in the brown planthopper. Among the pesticides shown to stimulate reproduction, jinggangmycin (JGM) is an unexpected example. JGM, a product of *Streptomyces var jinggangen*, is used in the control of rice sheath blight (RSB; an important disease of rice) caused by *Rhizoctonia sollani* in China (Peng et al. 2014). This fungicide is commonly applied two or three times during growing seasons, through leaf spraying at commercial rates of 125–175 g.a.i ha^−1^. In rice fields, RSB typically co-occurs with the brown planthopper. Thus, the study of the JGM-induced stimulation of brown planthopper reproduction has important implications for harmonious control of rice pests.

JGM stimulates reproduction in the brown planthopper, *N. lugens* Stål (Wu et al. 2001, Jiang et al. 2012), which is an issue worthy of concern because JGM is commonly overused given its low toxicity, lack of environmental residue, and low cost. Overuse of this antibiotic may result in an agricultural disaster in the form of uncontrollable rice pest resurgence, such as resurgence of the brown planthopper. Previous reports of pesticide-induced resurgence of the brown planthopper or mechanisms stimulating its reproduction have mainly involved insecticides (Reissig et al. 1982; Gao et al. 1988; Wang et al. 1994, 2010; Yin et al. 2008; Ge et al. 2010a, 2011) and of fungicide-induced resurgence of insect pests are relatively less. Some pesticides stimulate reproduction of other species of planthopper guild besides brown planthopper. For example, triazophos (TZP) stimulates the white-backed planthopper, *Sogatella furcifera* Horvath, and the small brown planthopper, *Laodelphax striatella* Fallen (Zhang et al. 2014). In addition, pesticides induce changes of stress in arthropod pest species (Guedes et al. 2016). For example, TZP enhanced thermotolerance of brown planthopper (Ge et al. 2013).

Recent studies demonstrate that the stimulation of brown planthopper reproduction induced by JGM is regulated by the fatty acid synthase (*FAS*) and acetyl-CoA carboxylase (*ACC*) genes (Zhang et al. 2015, Li et al. 2016). FAS is a large homodimeric enzyme with seven active sites necessary for the repeated, cyclical addition of acetyl moieties to growing fatty chains (Li et al. 2016). Animals obtain fatty acids in their food streams and produce saturated and monounsaturated fatty acids via FAS-catalyzed de novo synthesis. RNAi knockdown of FAS significantly decreases the fatty acid content of the ovary and fat body of the brown planthopper (Li et al. 2016). However, the effect of JGM on oocytes in the brown planthopper ovary is not clear. The changes in the oocytes of brown planthopper females treated with JGM have attracted attention because the development of oocytes is directly related to fecundity. Therefore, we observed the structure of oocytes in ovaries of females treated with JGM via transmission electron microscopy (TEM). In addition, oocyte maturation is regulated by hormones such as juvenile hormone (JH) and 20-hydroxyecdysone (20 E). Thus, we measured changes of JH and 20E levels.

## Materials and Methods

### Rice Variety, Insects, and JGM

The rice (*Oryza sativa* L.) variety Yangjing 805 (japonica rice) was used for each trial because this variety of rice is commonly planted in Jiangsu Province, China. The seeds were sown outdoors in standard rice-growing soil in cement tanks (height 60 cm, width 100 cm, and length 200 cm). When the seedlings reached the 6-leaf stage, they were transplanted into plastic pots (25-cm diameter, 35-cm height), with 4 hills per pot and 3 plants per hill. The rice plants used in the experiments were at the tillering stage.

The brown planthoppers used in the experiments were obtained from a stock population maintained at the China National Rice Research Institute (CNRRI; Hangzhou, China). The brown planthoppers were reared in an insect nursery on rice plants that were covered with cages. The insects were reared under natural conditions in cement tanks at Yangzhou University from April to October and in a greenhouse in the winter. Prior to the experiments, to ensure a sufficiently large population for release, two generations of growth were allowed to occur in the brown planthopper colony in an insectary with a controlled temperature and photoperiod (28 ± 2°C and a photoperiod of 14:10 [L:D] h), without insecticide application. Technical grade JGM (C_20_H_35_O_13_N; 61.7%) was supplied by Qianjiang Biochemical Co. Ltd. (Haining, Zhejiang, China).

### Spray Treatment

In total, 200 third-instar brown planthoppers per pot were released onto the rice plants at the tillering stage. Third-instar nymphs were used because many studies have demonstrated that these nymphs represent the key stage for pesticide-induced stimulation of reproduction in the brown planthopper (Yin et al. 2008, Jiang et al. 2012). Twenty-four hours after insect release, the potted plants were sprayed with 200 ppm JGM diluted with tap water (because JGM is water-soluble) after the addition of 10% emulsifier (ABS-Ca, an adjuvant; Jinglin Chemical Co. Ltd., Nanjing, Jiangsu, China) to reduce surface tension on the leaves. Control plants at the same developmental stage as the treated plants were sprayed with tap water and the nonactive substance (emulsifier). The treated and control plants were then covered with cages (screen size: 60 mesh). Each treatment was replicated thrice (3 pots), and the plants were distributed in a randomized pattern. The nymphs developed to the final fifth-instar stage (48 h after spray) on both the treated and control plants. The nymphs were collected and placed in glass jars (diameter 5 cm, height 10 cm) with untreated rice plants. The adult females were collected 1 and 2 d after emergence (1 and 2 DAE) to dissect the ovaries of adult females and to measure hormone levels.

### TEM Observation of the Ovaries

Fifteen adult females at 1 and 2 DAE were soaked in 0.1 mol liter^−1^ phosphate buffer solution and placed in a 4°C refrigerator. Three days later, the ovaries of the females were dissected and placed in a test tube, followed by the addition of 2.5% glutaraldehyde (0.1 mol liter^−1^ phosphate buffer solution, pH 7.3). The tubes were stored in a 4°C refrigerator. Ovary samples were fixed in 2.5% glutaraldehyde at 4°C for over 2 h, after which the fixative solution was discarded, and the samples were washed three times with phosphate buffer solution (pH 7.3). The samples were then fixed with 1% osmium tetroxide for 2 h, rewashed thrice with phosphate buffer solution (PBS), dehydrated with graded concentrations of ethanol (50, 70, 80, 90, and 100%), and washed with acetone. After soaking with a 1:1 mixture of acetone and resin for 1 h, the samples were soaked with a 1:2 mixture of acetone and resin for 2 h and embedded with pure resin and sliced using an EMUC6 frozen ultramicrotome (Leica Microsystems Ltd. Co., Germany). The slices were observed and photographed with a Tecnai 12 TEM (Philips-FEI Co. Ltd., Holland) after staining with uranyl acetate and lead acetate. The size of lipid droplets in the oocytes was measured with Photoshop.

### Measurement of JH and 20 E Levels in Females

JH and 20 E regulate the reproduction of insects. Therefore, we measured changes in these hormones. JH and 20 E levels were quantified via enzyme-linked immunosorbent assay (ELISA) according to method of Spearow (1987). ELISA kits for JH and 20 E were provided by the Shanghai QiaoDu Biotech Co., Ltd. Female specimens were rapidly frozen with liquid nitrogen after the addition of 0.01 mol PBS (0.24 g KH_2_PO_4_ + 1.44 g Na_2_HPO_4_ + 8.0 g NaCl +0.2 g KCl + 800 ml water, adjusted pH to 7.4 with HCl and HaOH, brought to 1 liter with water). The samples were then ground with a homogenizer after the addition of 0.01 mol PBS and centrifuged at 3,000 rpm for 20 min. Then, the supernatant was collected. The process of measuring JH and 20 E involved the following steps: 1) dilution of the JH or 20 E standard and sample addition (10 standard wells were arranged in a microtitration plate; 100 µl of the JH or 20 E standard was added to the first and second wells after the addition of 50 µl of a standard dilution solution and evenly shaken; 100 µl of the solution from the first and second wells was then added to the third and fourth wells after the addition of 50 µl of a standard dilution solution and evenly shaken, and so on; and finally, 50 µl of the solution from the 9th and 10th wells was removed). 2) Sample addition: control (without sample or standard sample addition) and sample wells were arranged, and 10 µl of a sample solution was added after 40 µl of a standard dilution solution and evenly shaken. 3) Incubation: plates covered with plate closure membranes were incubated at 37°C for 30 min. 4) Washing: the membrane was uncovered; the solution was removed; washing liquid (provided by ELISA kits) was added and removed after 30 s; and this process was repeated five times. 5) Enzyme addition: 50 µl of ELISA reagents was added per well (except for the control wells) followed by incubation and washing; this procedure was repeated once. 6) Color development: 50 µl of coloring reagent A (provided in the ELISA kits) was added to each well; 50 µl of coloring reagent B (provided in the ELISA kits) was added, followed by even shaking and development at 37°C for 15 min in the dark; and finally, 50 µl of stop solution was added per well (from blue to yellow). 7) Measurements: the absorbance value (OD) at 450 nm was detected using a microplate reader (Labsystems Multiskan MS, Model 352, Finland).

### Statistical Analyses

The normality of the distribution and the homogeneity of variance (determined using the Bartlett test) were verified prior to performing analysis of variance (ANOVA). Two-way ANOVAs (JGM treatment and days after adult emergence (DAE)) were conducted for the size of lipid droplets in oocytes and the levels of JH and 20 E. Multiple comparisons of means were performed using Fisher’s protected least-significant-difference tests. All analyses were conducted using the data processing system of Tang et al. (2002).

## Results

### Effect of JGM on Ovary Development in Adult Females

**Fig. 1.**
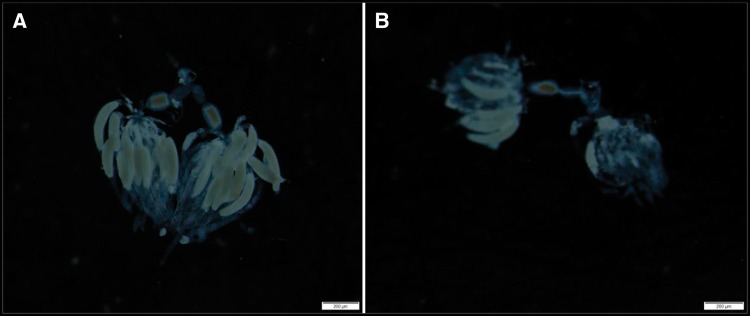
Brown planthopper ovaries at 2 DAE for JGM and control. **A**: JGM treatment, **B**: Control, DAE: days after emergence.

Ovary development occurred faster in JGM-treated females than in the control females, and the resultant number of mature oocytes was also increased compared with that of the control females (Fig. 1).

### Changes in the Size of Lipid Droplets in the Oocytes of JGM-Treated Females

**Fig. 2.**
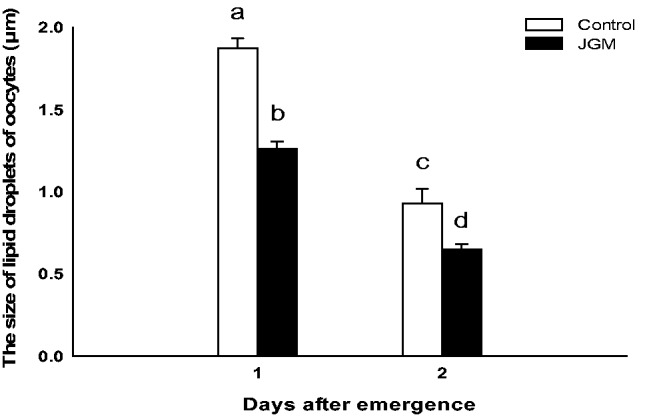
The size of lipid droplets in oocytes in the ovaries of JGM-treated and control brown planthopper females. The bars of the histogram represent the values indicated on the Y-axes, and the error bars represent the standard error of the mean (SEM). Bars annotated with the same letter within the same day after emergence are not significantly different at the 5% level.

**Fig. 3.**
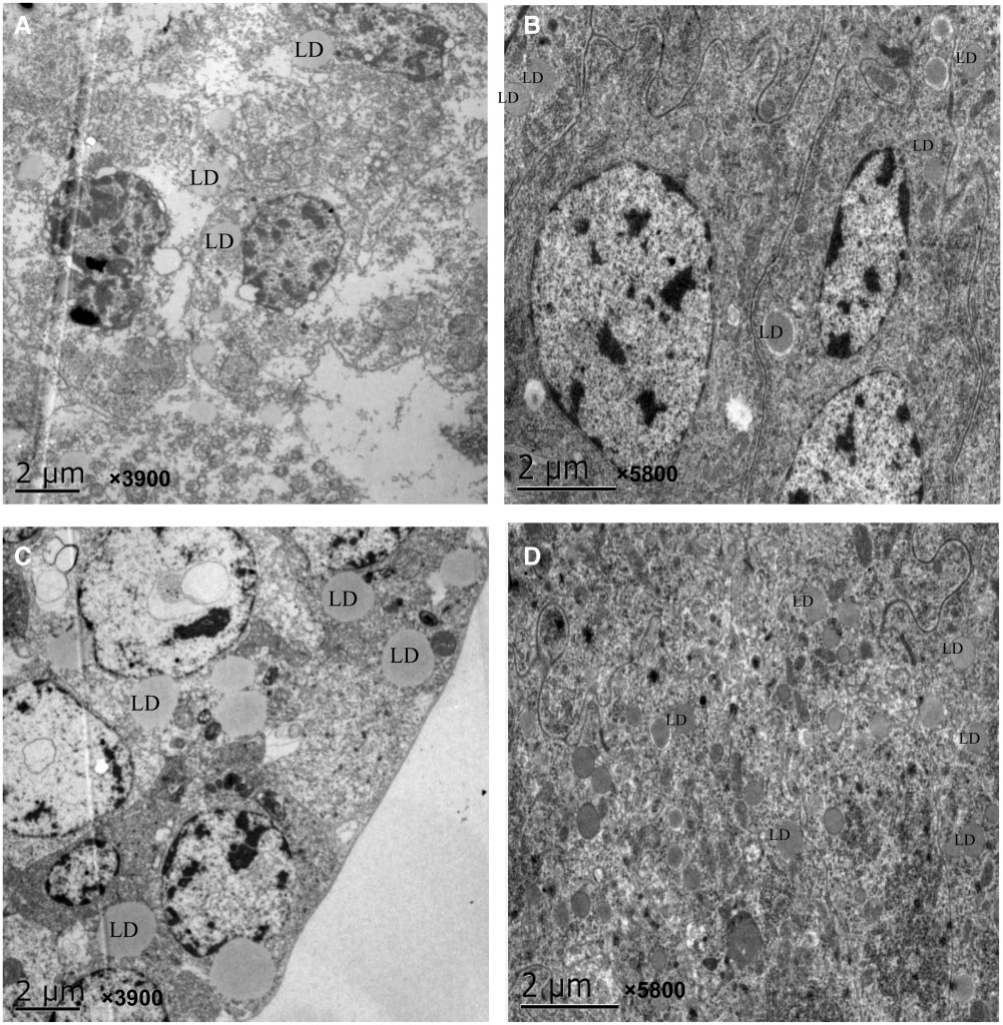
Oocytes in the ovaries of JGM-treated and control females at 1 and 2 DAE. LD is lipid droplets. **A**: 1 DAE for JGM treatment, **B**: 2 DAE for JGM treatment, **C**: 1 DAE for the control, **D**: 2 DAE for the control.

The size of lipid droplets in the oocytes of JGM-treated females was significantly smaller than that of the control females (*F* = 53.5; df = 1, 32; *P* = 0.0001; Figs 2 and 3). The diameter of lipid droplets (DLD) at 1 and 2 DAE following JGM treatment was decreased by 32.6 and 29.8%, respectively. A significant effects of DAE and the interaction between JGM and DAE on the DLD (*F* = 164.9; df = 1, 32; *P* = 0.0001 for DAE; *F* = 7.6; df = 1, 32; *P* = 0.01 for interaction effect) were noted. The DLD at 2 DAE, compared with that at 1 DAE, was significantly reduced by 50.5 and 48.4% in the JGM and control groups, respectively.

### Effect of JGM on JH and 20 E Levels

**Fig. 4.**
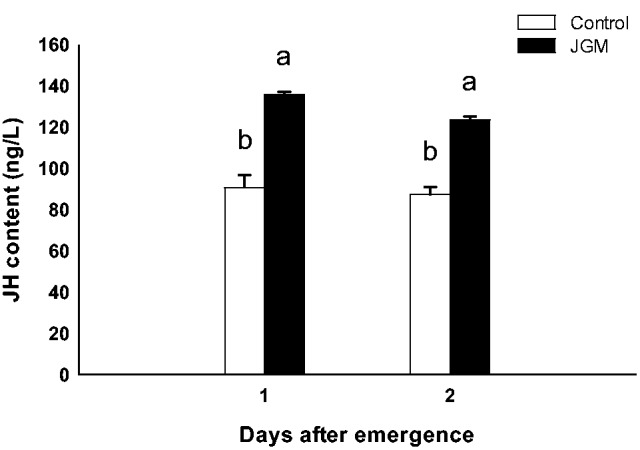
JH levels in JGM-treated and control adult females. The bars of the histogram represent the values indicated on the Y-axes, and the error bars represent the SEM. Bars annotated with the same letter within the same day after emergence are not significantly different at the 5% level.

**Fig. 5.**
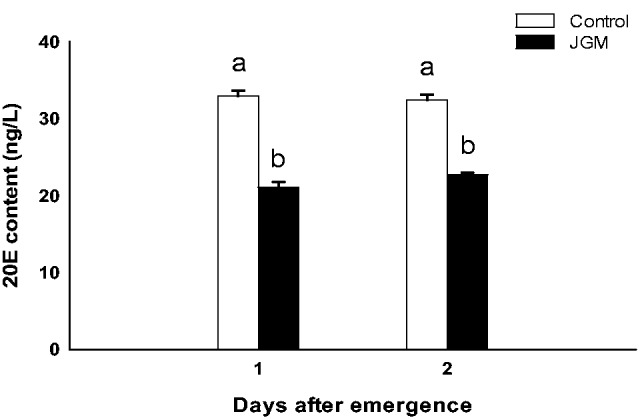
20E levels in JGM-treated and control adult females. The bars of the histogram represent the values indicated on the Y-axes, and the error bars represent the SEM. Bars annotated with the same letter within the same day after emergence are not significantly different at the 5% level.

JH levels in JGM-treated females, compared with those in the control females, were significantly increased (*F* = 100.4; df = 1, 8; *P* = 0.0001; Fig. 4). JH levels at 1 and 2 DAE under JGM treatment increased by 49.7 and 45.7%, respectively, compared with the control. No significant effects of DAE and the interaction between JGM and DAE on JH levels were noted (*F* = 2.0; df = 1, 8; *P* = 0.19 for DAE; *F* = 0.38; df = 1, 8; *P* = 0.55 for interaction effect). In contrast to JH levels, 20 E levels in JGM-treated females, compared with those in control females, were significantly reduced by 36.0 and 30.0% at 1 and 2 DAE, respectively, (*F* = 309.6; df = 1, 8; *P* = 0.0001; Fig. 5). No significant effects of DAE and the interaction between JGM and DAE on 20 E levels were noted (*F* = 0.67; df = 1, 8; *P* = 0.44 for DAE; *F* = 3.0; df = 1, 8; *P* = 0.11 for interaction effect).

## Discussion

Isobaric tags for relative and absolute quantitation (iTRAQ) protocols applied to JGM-treated females have demonstrated that the expression of several proteins, such as cell division control-related protein 1, fatty acid synthase (FAS), acyl-CoA-binding protein, and cell division cycle protein 37, is up-regulated (Li et al. 2016). Measurement of fatty acids revealed significant increases in several acids in JGM-treated females; dsFAS reduces the fecundity of JGM-treated females and the concentrations of several fatty acids in the ovary and fat body (Li et al. 2016). The TEM observations performed in the present study revealed that lipid droplets were significantly reduced in the oocytes of JGM-treated females compared with those in the oocytes of the control females, indicating that JGM promotes metabolism of fatty acids.

The biological roles of insect fatty acids are highly significant and diverse. Fatty acids are associated with reproduction in insects (Thiery et al. 1995, Kamoshida et al. 2012). Fatty acids enter the well-known energy storage and production pathways. In addition to homeostatic metabolism, fatty acids support flight, reproduction, and long-term diapause and estivation. Fatty acids are also involved in alternative pathways unrelated to energy metabolism. These pathways change the structure of the fatty acids and carry them from one area of biological significance to others, including roles as waxes, pheromones, eicosanoids, and components of defensive secretions (Stanley-Samuelson et al. 1988, Watts et al. 2003, Sayah 2008, Alabaster et al. 2011, Vrablik and Watt 2012). Among various biochemical processes, the β-oxidation of fatty acids plays specific physiological and biochemical roles in addition to its role in supplying energy for insects or other animals. For example, β-oxidation of lipids is required for the generation of oocytes in mice. Moreover, the addition of L-carnitine during ovarian follicle development in vitro increases lipid metabolism and improves oocyte development (Dunning et al. 2011). Such observations of the cytology of oocytes verify that FAS regulates the JGM-induced stimulation of brown planthopper reproduction.

Insect hormones regulate the growth and development and fecundity of insects (Koolman 1989, Comas et al. 1999, Rauschenbach et al. 2001, Wang 2004) and control egg laying and guarding in the lace bug (Tallamy et al. 2002). Additionally, insect hormones can induce acetylcholinesterase activity in a *Drosophila* cell line (Cherbas 1977). Among these hormones, JH regulates the synthesis of vitellogenin (Vg). JH stimulates Vg biosynthesis in female fat body and follicle cells and stimulates uptake by oocytes from the hemolymph in growing follicles (Nation 2002). Thus, we measured the changes in JH and 20 E levels. 20 E regulates the fecundity of adult insects in concert with JH (Ye et al. 1999, Wang 2004). Ecdysterone induces two activities (acetylcholinesterase and β-galactosidase) of cell lines of *Drosophila melanogaster* (Best-Belpomme & Courgeon, 1980). Thus, the continuous presence of a subthreshold concentration of ecdysterone provokes the maturation of the cell which become able to respond to the hormonal stimulus by a quicker and higher enzymatic induction (Best-Belpomme & Courgeon, 1980). However, altosid (a juvenile hormone analog) abolishes the effects of the ecdysterone-induced maturation (Best-Belpomme & Courgeon, 1980). This shows that JH or 20 E regulates growth and development of insect via metabolism. Studies show that molecular mechanisms of pesticide-induced stimulation of reproduction of brown planthopper are also involved in metabolic pathway (Jiang et al. 2016, Zhang et al. 2015, Li et al. 2016). In this context, the hormonal changes in insects after pesticide application are very interesting. This study showed that JGM enhanced JH levels in brown planthopper females but reduced 20 E levels. In adult females, JH promote fecundity. However, the relationship between increased JH titers in adult females after insecticide treatment and fecundity depends on the insect species. JH titers in adult female *Tryporyza incertulas* Walker developed from larvae feeding on rice plants treated with imidacloprid were significantly increased, and the number of eggs laid by these females was also significantly increased (Wang et al. 2005). However, *Chilo suppressalis* (Walker) does not exhibit a consistent trend between the increase in JH titers and the fecundity of female adults after foliar spraying imidacloprid (Yu et al. 2007). Some insecticides, such as triazophos and deltamethrin, enhance JH titers and Vg mRNA expression levels, while suppressing JH esterase (JHE) expression levels (Ge et al. 2010b). JHE potentially plays a key role in the metabolism of JH (de Kort and Granger 1996). The tested insecticide suppressed JHE expression, enhanced JH levels, and resulted in an increase of fecundity in brown planthopper females.

## References

[tow085-B1] Alabaster A. Isoe J. Zhou G. L. Lee A. Murphy A. Day W. A. Miesfeld. R. L. 2011 Deficiencies in acetyl-CoA carboxylase and fatty acid synthase 1 differentially affect eggshell formation and blood meal digestion in *Aedes aegypti*, Insect Biochem. Mol. Biol. 41: 946–955. 10.1016/j.ibmb.2011.09.004 PMC3210400 21971482

[tow085-B3] Cherbas P. Cherbas L. Williams. C. M. 1977 Induction of acetylcholinesterase activity by β-Ecdysone in a *Drosophila* cell line. Sci. (Wash. D.C.) 197: 275–277. 10.1126/science.877552 877552

[tow085-B4] Comas D. Piulachs M. D. Belles. X. 1999 Fast induction of vitellogenin gene expression by juvenile hormone III in the cockroach *Blattella germanica* (L) (Dictyoptera: Blattellidae). Insect Biochem. Mol. Biol. 29: 821–827. 10510500 10.1016/s0965-1748(99)00058-2

[tow085-B5] de Kort C.A.D. Granger. N. A. 1996 Regulation of JH titers: The relevance of degradative enzymes and binding proteins. Arch. Insect Biochem. Physiol. 33: 1–26.

[tow085-B6] Dunning K. R. Akison L. K. Russell D. L. Norman R. J. Roker. R. L. 2011 Increased beta-oxidation and improved oocyte development competence to L-carnitine during ovarian in vitro follicle development in mice. Biol. Reprod. 85: 548–555. 21613630 10.1095/biolreprod.110.090415

[tow085-B7] Gao C. X. Gu X. H. Bei Y. W. Wang. R. M. 1988 Approach of causes on brown planthopper resurgence. Acta Ecol. Sin. 8: 155–163.

[tow085-B8] Ge L. Q. Wu J. C. Zhao K. F. Chen Y. Yang. G. Q. 2010a Induction of *Nlvg* and suppression of *Nljhe* gene expression in *Nilaparvata lugens* (Stål) (Hemiptera: Delphacidae) adult females and males exposed to two insecticides. Pesti. Biochem. Physiol. 98: 269–278.

[tow085-B9] Ge L. Q. Wang L. P. Zhao K. F. Wu J. C. Huang. L. J. 2010b Mating pair combinations of insecticide-treated male and female *Nilaparvata lugens* Stål (Hemiptera: Delphacidae) planthoppers influence protein content in the male accessory glands (MAGs) and vitellin content in both fat bodies and ovaries of adult females. Pestic. Biochem. Physiol. 98: 279–288.

[tow085-B10] Ge L. Q. Cheng Y. Wu J. C. Jahn. G. C. 2011 Proteomic analysis of insecticide triazophos-induced mating-responsive proteins of *Nilaparvata lugens* Stål (Hemiptera: Delphacidae). J. Proteome Res. 10: 4597–4612. 21800909 10.1021/pr200414g

[tow085-B11] Ge L. Q. Huang L. J. Yang G. Q. Song Q. S. Stanley D. Gurr G. M. Wu. J. C. 2013 Molecular basis for insecticide-enhanced thermotolerance in the brown planthopper *Nilaparvata lugens* Stål (Hemiptera: Delphacidae). Mol. Ecol. 22: 5624–5634. 24303791 10.1111/mec.12502

[tow085-B12] Guedes R.N.C. Smagghe G. Stark J. D. Desneux. N. 2016 Pesticide-induced stress in arthropod pests for optimized integrated pest management programs. Ann. Rev. Entomol. 61: 3.1–3.20. 10.1146/annurev-ento-010715-023646 26473315

[tow085-B13] Jiang L. B. Zhao K. F. Wang D. J. Wu. J. C. 2012 Effects of different treatment methods of the fungicide jinggangmycin on reproduction and vitellogenin gene (*Nlvg*) expression in the brown planthopper *Nilaparvata lugens* Stål (Hemiptera: Delphacidae). Pestic. Biochem. Physiol. 102: 51–55.

[tow085-B14] Jiang Y. P. Li L. Liu Z. Y. Wu Y. Xu B. Ge L. Q. Song Q. S. Wu. J. C. 2016 Adipose triglyceride lipase (Atgl) mediates the antibiotic jinggangmycin-stimulated reproduction in the brown planthopper, *Nilaparvata lugens* Stål. Sci. Rep. 6: 18984 (DOI:10.1038/srep18984). 26739506 10.1038/srep18984 PMC4704046

[tow085-B15] Kamoshida Y. Fujiyama-Nakamura S. Kimura S. Suzuki E. Lim J. Shiozaki-Sato Y. Takeyama K. I. Kato. S. 2012 Ecdysone receptor (EcR) suppreses lipid accumulation in the *Drosophila* fat body via transcription control, Biochem. Biophy*.* Res. Communi*.* 421: 203–207. 10.1016/j.bbrc.2012.03.135 22503687

[tow085-B16] Koolman J. 1989 Ecdysone: from chemistry to mode of action. Gerog Thieme Verlag, Stuttgart, Germany.

[tow085-B18] Li L. Jiang Y. P. Liu Z. Y. You L. L. Wu Y. Xu B. Ge L. Q. Stanley D. Song Q. S. Wu. J. C. 2016 Jinggangmycin increases fecundity of the brown planthopper, *Nilaparvata lugens* (Stål) via fatty acid synthase gene expression. J. Proteomics 130: 140–149. 26388431 10.1016/j.jprot.2015.09.022

[tow085-B19] Nation J. L. 2002 Insect Physiology and Biochemistry. CRC Press, New York, pp. 425–451.

[tow085-B20] Peng D. Li S. Wang J. Chen C. Zhou. M. 2014 Integrated biological and chemical control of rice sheath blight by *Bacillus subtilis*. Pesti. Biochem. Physiol. 102: 258–263. 10.1002/ps.3551 23564744

[tow085-B21] Rauschenbach I. Yu N. Gruntenko E. Chentsova N. S. Hirashima A. Sukhanova Zh. Andreenkova E. V. Glazko. G. V. 2001 Regulation of reproductive function in *Drosophila* females due to hormonal interaction under stress is genetically determined. Russ. J. Genet. 37: 1041–1047. 11642127

[tow085-B22] Reissig W. H. Heinrichs E. A. Valencia. S. L. 1982 Effects of insecticides on *Nilaparvata lugens* and its predators: spider, *Microvelia atrolineata*, and *Cyrtorhinus lividipennis*. Environ. Entomol. 11: 193–199.

[tow085-B23] Sayah F. 2008 Changes in the lipid and fatty acid composition of hemolymph and ovaries during the reproductive cycle of *Labidura riparia*, Entomol. Sci. 11: 55–53.

[tow085-B24] Spearow J. L. Trost. B. A. 1987 Development of a sensitive enzyme-linked immunosorbent assay for cattle, sheep, rat, and mouse luteinizing hormone. Biol. Reprod. 37: 596–605. 10.1095/biolreprod37.3.595 2823922

[tow085-B25] Stanley-Samuelson D. W. Jurenka R. A. Cripps C. Blomquist G. J. de Renobales. M. 1988 Fatty acids in insects: Composition, metabolism and biological significance, Arch. Insect Biochem. Physiol. 9: 1–33.

[tow085-B26] Tallamy D. W. Monaco E. L. Pesek. J. D. 2002 Hormone control of egg dumping and guarding in the lace bug, *Cargaphia solani* (Hemiptera: Tingidae). J. Insect Behav. 15: 467–475.

[tow085-B27] Tang Q. Y. Feng M. G. 2002 DPS data processing system for practical statistics. Scientific Press, Bejing, China.

[tow085-B28] Thiery D. Gabel B. Farkas P. Jarry. M. 1995 Egg dispersion in codling moth: Influence of egg extract and of its fatty acid constituents, J. Chem. Ecol. 21: 2015–2026. 24233903 10.1007/BF02033859

[tow085-B29] Vrablik T. L. Watt. J. L. 2012 Emerging roles for specific fatty acids in developmental processes, Genes Dev. 26: 631–637. 22474257 10.1101/gad.190777.112 PMC3323873

[tow085-B30] Wang Y. C. 2004 Insect Physiology. China Agricultural Press, Bejing, China.

[tow085-B31] Wang Y. C. Fang J. Q. Tian X. Z. Gao B. Z. Fan. Y. R. 1994 Studies on the resurgent question of planthoppers induced by deltamethrin and methamidophos. Entomol. Knowl. 31: 257–262.

[tow085-B32] Wang A. H. Wu J. C. Xu J. F. Yang G. Q. Qiu H. M. Li. D. H. 2005 Selective insecticide-induced stimulation on fecundity and biochemical changes in *Tryporyza incertulas* (Lepideptera: Pyralidae). J. Econ. Entomol. 98: 1144–1149. 16156564 10.1603/0022-0493-98.4.1144

[tow085-B33] Wang L. P. Shen J. Ge L. Q. Wu J. C. Yang G. Q. Jahn. G. C. 2010 Insecticide-induced increase in the protein content of male accessory glands and its effect on the fecundity of females in the brown planthopper *Nilaparvata lugens* Stål (Hemiptera: Delphacidae). Crop Prot. 29: 1280–1285.

[tow085-B34] Watts J. L. Phillips E. Griffing K. R. Browse. J. 2003 Deficiencies in C20 polyunsaturated fatty acids cause behavioral and developmental defects in *Caenorhabditis elegans* fat-3 mutants, Genetics. 163: 581–589. 12618397 10.1093/genetics/163.2.581 PMC1462460

[tow085-B35] Wu J. C. Xu J. X. Yuan S. Z. Liu J. L. Jiang Y. H. Xu. J. F. 2001 Pesticide-induced susceptibility of rice to brown planthopper *Nilaparvata lugens*. Entomol. Exp. Appl. 100: 119–126.

[tow085-B36] Ye G. Y. Hu C. Gong. H. 1999 Effects of juvenile hormone and ecdysteroid on ovarian development of the Japanese oak silkworm *Antheraea yamamai*. J. Zhejiang Agric. Univ. 25: 276–280.

[tow085-B37] Yin J. L. Xu H. W. Wu J. C. Hu J. H. Yang. G. Q. 2008 Cultivar and insecticide applications affect the physiological development of the brown planthopper, *Nilaparvata lugens* (Stål) (Homoptera: Delphacidae). Environ. Entomol. 37: 206–212. 18348812 10.1603/0046-225x(2008)37[206:caiaat]2.0.co;2

[tow085-B38] Yu Y. S. Xue S. Wu J. C. Wang F. Liu J. L. Gu. H. N. 2007 Distribution of imidacloprid in different parts of rice plants and its impact on juvenile hormone and molting hormone in larvae of a non-target insect the stripe stem borer, *Chilo suppresslis* (Walker) (Lepidoptera: Pyralidae). J. Econ. Entomol. 100: 375–380. 17461061 10.1603/0022-0493(2007)100[375:doirid]2.0.co;2

[tow085-B39] Zhang Y. X. Zhu Z. F. Lu X. L. Li X. Ge L. Q. Fang J. C. Wu. J. C. 2014 Effects of two pesticides, TZP and JGM, on reproduction of three planthopper species, *Nilaparvata lugens* Stål, *Sogatella furcifera* Horvath, and *Laodelphax striatella* Fallen. Pesti. Biochem. Physiol. 115: 53–57. 10.1016/j.pestbp.2014.07.012 25307466

[tow085-B40] Zhang Y. X. Ge L. Q. Jiang Y. P. Lu X. L. Li X. Stanley D. Song Q. S. Wu. J. C. 2015 RNAi knockdown of acetyl-CoA carboxylase gene eliminates jinggangmycin-enhanced reproduction and population growth in the brown planthopper, *Nilaparvata lugens*. Sci. Rep. 5: 15360 (DOI:10:1038/srep15360). 26482193 10.1038/srep15360 PMC4611885

